# Supplementary material for: Uncovering hidden protein modifications with native top-down mass spectrometry
Source: Nat Methods. 2025 Sep 29;22(10):2127–37. doi: 10.1038/s41592-025-02846-5 (PMC12510877; doi:10.1038/s41592-025-02846-5)
Supplement: Supplementary file 1 — Supplementary Figs. 1–20, Tables 1 and 2 and Notes 1–3. [file 41592_2025_2846_MOESM1_ESM.pdf]

nature methods

Supplementary information

https://doi.org/10.1038/s41592-025-02846-5

Uncovering hidden protein modifications with native top-down mass spectrometry

In the format provided by the authors and unedited

Table of Contents

Supplementary Figures 2

Supplementary Figure 1: Peak picking and reconstruction of high-resolution Orbitrap mass spectra using a modified Richardson-Lucy algorithm 2

Supplementary Figure 2: Performance of the supervised voting classifier in minimizing and balancing errors in isotopic envelope identification 3

Supplementary Figure 3: Reducing mass errors with spectral recalibration 4

Supplementary Figure 4: precisION's fragment-level open search enables comprehensive spectral assignment 5

Supplementary Figure 5: Identifying sets of internal fragments with a shared terminal fragmentation site using a multinotch fragment-level open search 6

Supplementary Figure 6: precisION's multinotch enables highly effective filtering of putative internal fragments 7

Supplementary Figure 7: Impact of the supervised voting envelope classifier on the fragment-level search 8

Supplementary Figure 8: Influence of the mass accuracy threshold on the fragment-level open search 19

Supplementary Figure 9: Higher-energy collisional dissociation results in extensive fragmentation across the length of SPP1 10

Supplementary Figure 10: Sequence ions generated by higher-energy collisional dissociation map the entire length of human SPP1 11

Supplementary Figure 11: A fragment-level open search reveals diverse N-terminal modification of SPP1 12

Supplementary Figure 12: Relative occupancy of different phosphate stoichiometries along SPP1 13

Supplementary Figure 13: Electron transfer dissociation confirms low phospho-occupancy of SPP1 termini 14

Supplementary Figure 14: Infrared multiphoton dissociation generates fragments along the length of GAT1 15

Supplementary Figure 15: Human GAT1 lipidation can be observed and reproducibly modelled across sequence ions and spectra 16

Supplementary Figure 16: Human GAT1 lipidation is consistent across replicate expressions 17

Supplementary Figure 17: Internal fragments in nTDMS primarily form via bond cleavage at high-propensity fragmentation sites 18

Supplementary Figure 18: True and false fragment ions can be partially distinguished on the basis of their isotopic envelope fit 19

Supplementary Figure 19: precisION correctly identifies misassignments in entrapment-style evaluations 20

Supplementary Figure 20: Internal fragment ion-level FDR calculations are supported by MS3 experiments 21

Supplementary Tables 22

Supplementary Table 1: List of features used to represent putative fragment ion envelopes 22

Supplementary Table 2: List of SPP1 MS/MS spectra used for the quantification of protein phosphorylation 23

Supplementary Notes 24

Supplementary Note 1: Analysis of endogenous multiprotein complexes - PDE6 complex from bovine retina 24

Supplementary Note 2: Entrapment-style evaluation and robustness assessment 25

Supplementary Note 3: Custom goodness-of-fit scoring methodology 26

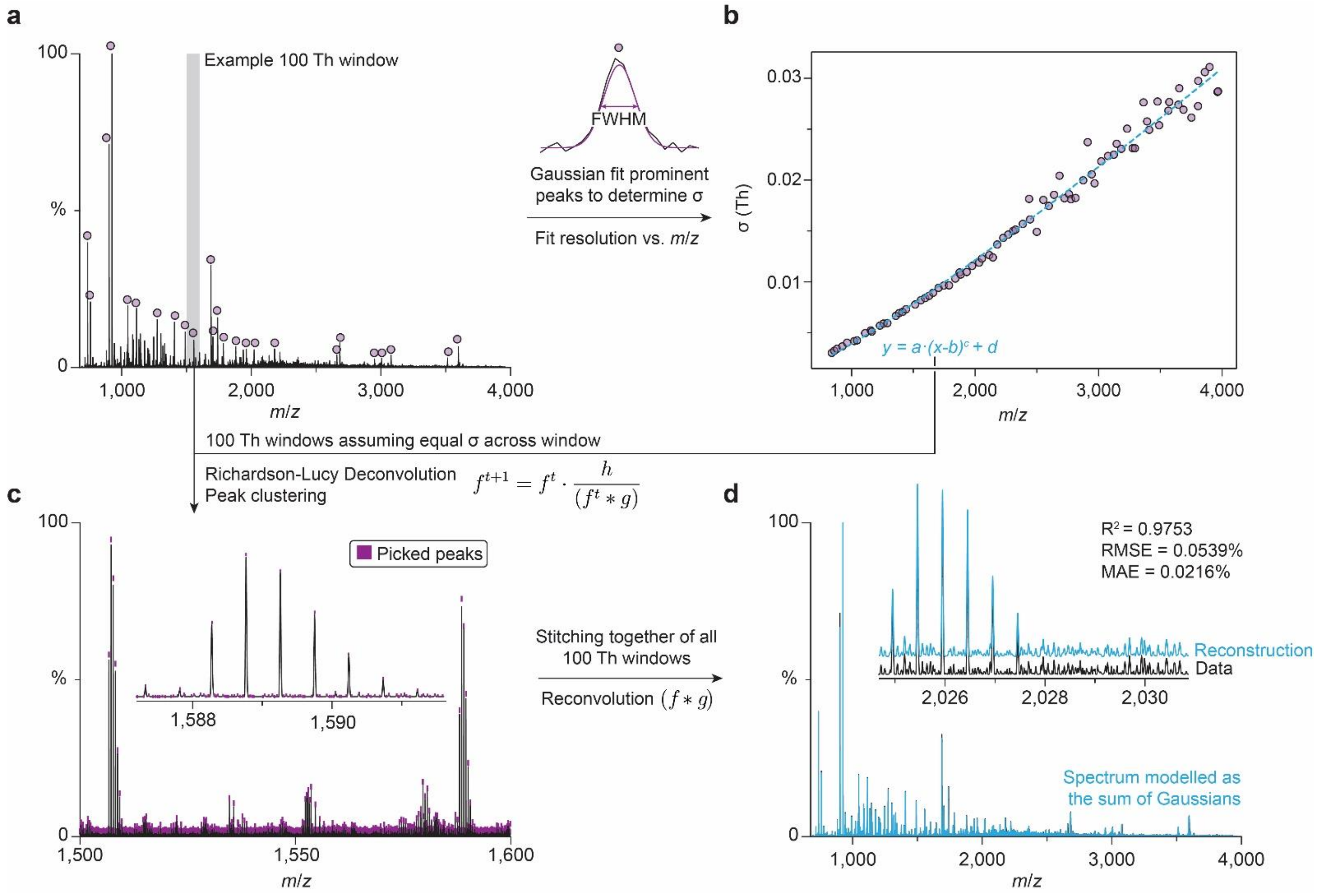

Supplementary Figure 1: Peak picking and reconstruction of high-resolution Orbitrap mass spectra using a modified Richardson-Lucy algorithm. Schematic illustrating the modified Richardson-Lucy algorithm implemented in precisION. **a**, High-intensity peaks from across the spectrum are identified and fit with a Gaussian point spread function (psf). **b,** The relationship between Gaussian peak width (σ) and *m*/*z* is modelled using a general power function. **c**, Peaks are picked from 100 Th windows of the raw spectrum using a modified Richardson-Lucy deconvolution algorithm (see **Methods** for further details). The value of σ for the Gaussian psf used in deconvolving each window is calculated using the previously defined power function. **d**, After all windows are deconvolved, the full spectrum is reconstructed by stitching all of the 100 Th windows together and reconvolving the resulting centroided spectrum with the *m*/*z*-dependent psf, resulting in a 'reconstructed' profile spectrum that closely matches the experimental data with reduced noise. The final peak list and reconstructed spectrum can be further analyzed to identify fragment ion envelopes.

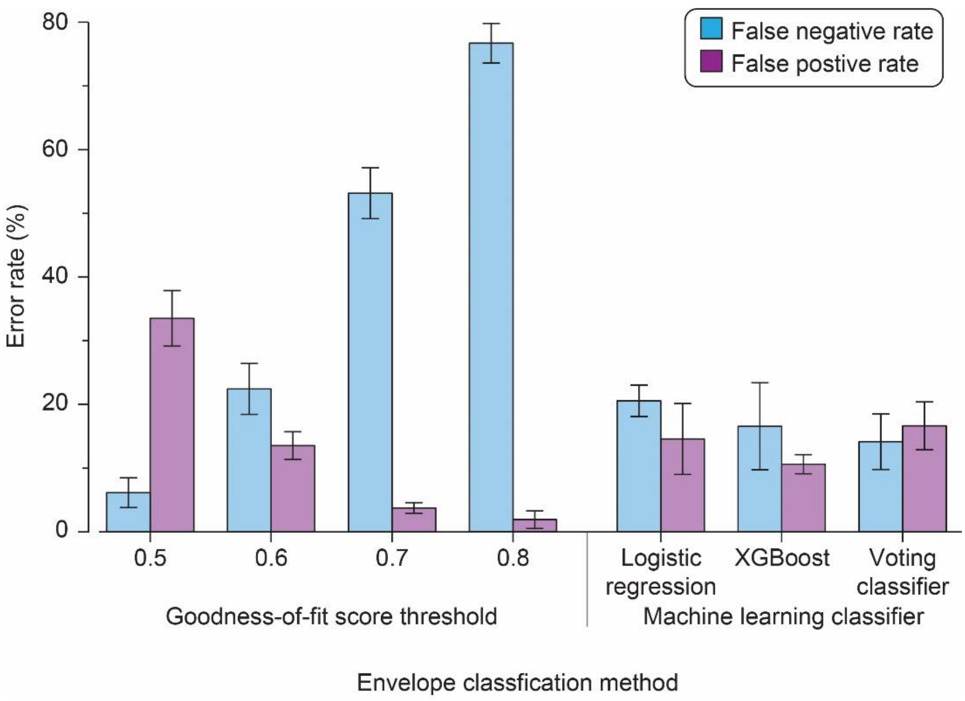

Supplementary Figure 2: Performance of the supervised voting classifier in minimizing and balancing errors in isotopic envelope identification. Comparison of the false negative rate (blue) and false positive rate (purple) across different isotopic envelope classifiers. Envelopes were identified from the HCD 130 V MS/MS spectrum of the ACE2 dimer using a combined deconvolution strategy, with additional filtering to remove envelopes with a signal-to-noise ratio below 3. A total of 856 envelopes (375 true, 481 false) out of 2,737 identified were manually classified by the author to form a 'ground truth' dataset that was used to train independent logistic regression and XGBoost classifiers. This set of 856 envelopes was used to evaluate the error rates of established classifiers (based on fixed goodness-of-fit thresholds), individual machine learning classifiers, and the precisION voting classifier. Five-fold cross-validation was used. The voting classifier demonstrates a reduced false negative rate compared to individual machine learning models, decreasing the number of true envelopes that are discarded. Furthermore, the supervised voting classifier exhibits balanced and low (<20%) false negative and false positive rates. Data are presented as the mean ± s.d. from five-fold cross-validation runs.

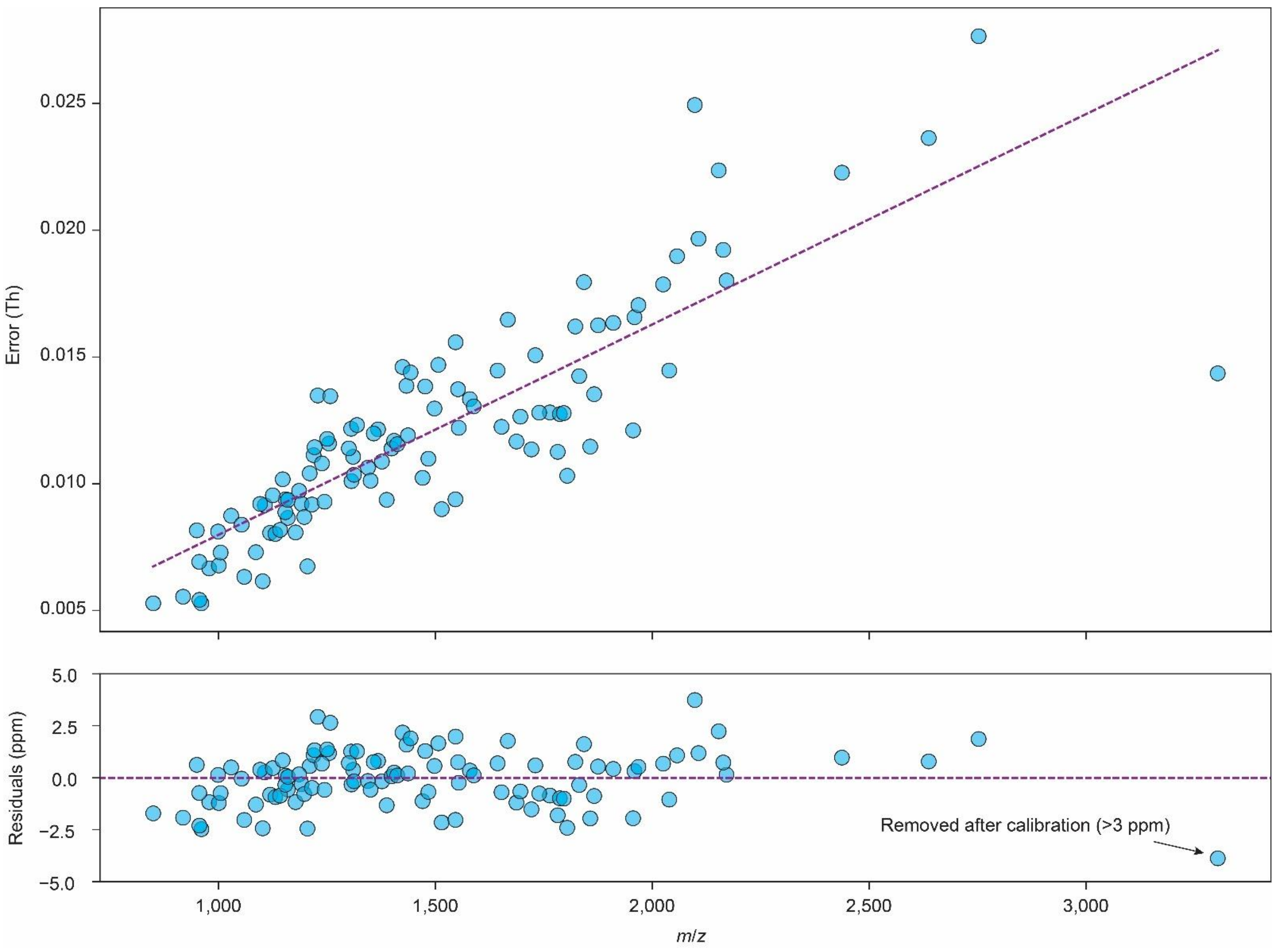

Supplementary Figure 3: Reducing mass errors with spectral recalibration. Unmodified terminal fragments and their gas-phase products (e.g., dehydration) are used to recalibrate mass spectra following an initial round of ion assignment with a broad mass tolerance of 10 ppm. The mass errors are modelled using a linear function, which corrects for mass analyzer drift and increases mass accuracy. Here, for an Orbitrap (R = 240,000 @ *m*/*z* 200), nearly all mass errors are reduced to less than 3 ppm after calibration. Assignments that do not satisfy a user-specified mass accuracy threshold are removed after calibration.

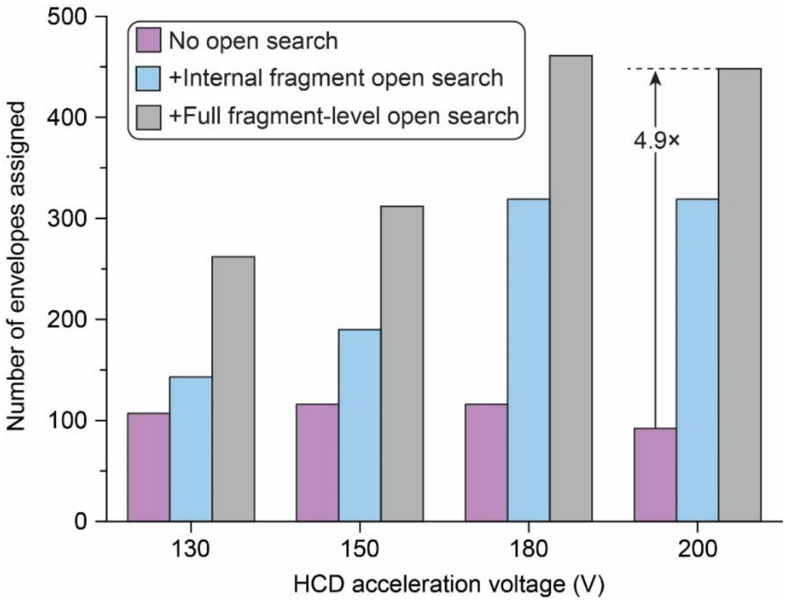

Supplementary Figure 4: precisION's fragment-level open search enables comprehensive spectral assignment. Number of envelopes assigned without the open search (purple), with the multinotch internal fragment open search (blue), and with the full fragment-level open search (grey) from HCD MS/MS spectra of the ACE2 dimer. In all cases, the open search greatly increases the number of isotopic envelopes that can be assigned to (modified) sequence ions.

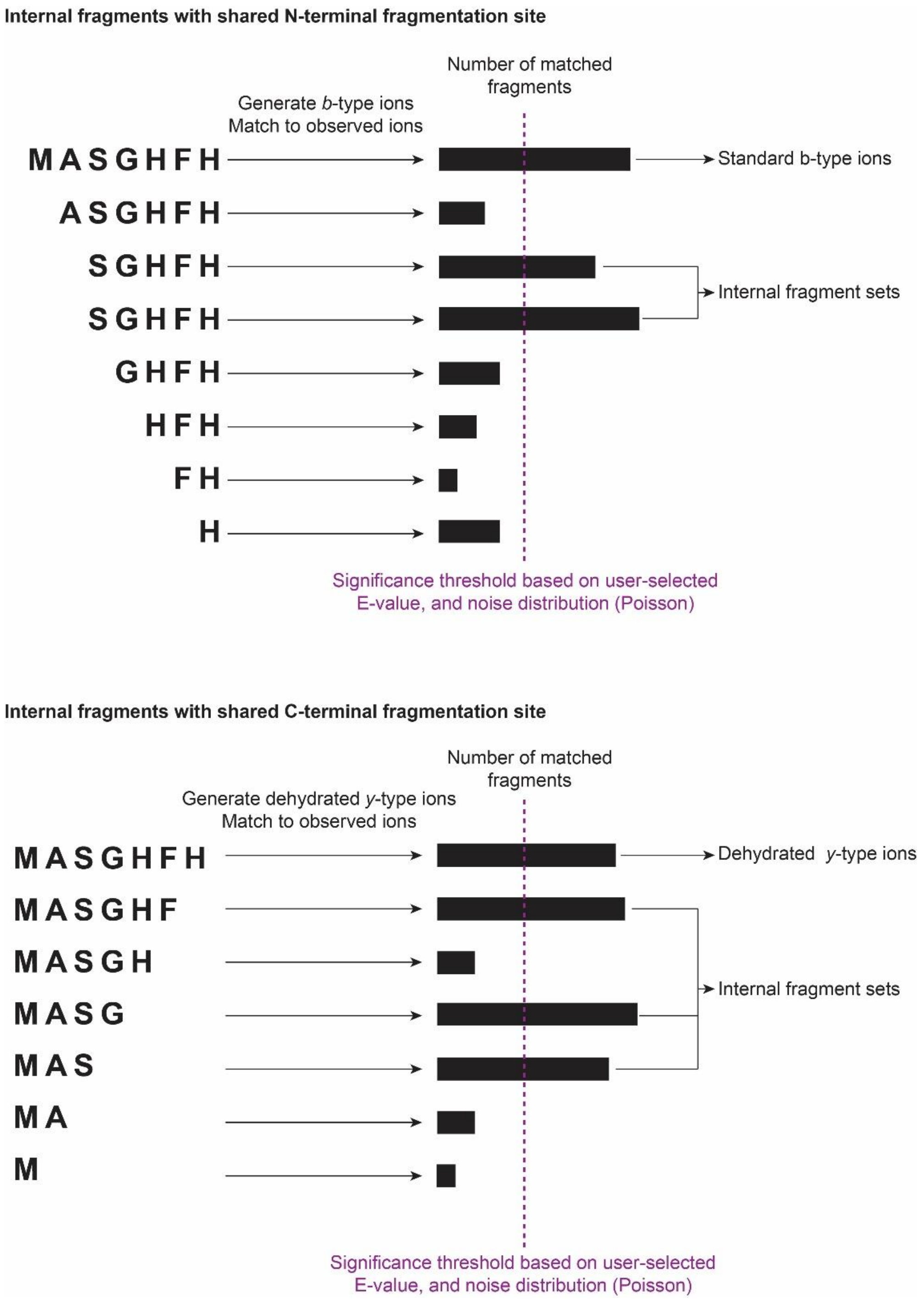

Supplementary Figure 5: Identifying sets of internal fragments with a shared terminal fragmentation site using a multinotch fragment-level open search. A modified fragment-level open search is used to identify sets of internal fragments sharing a common terminal fragmentation site, where the size of the sets exceeds what would be expected from random matching. The search examines offsets corresponding to sequential losses of individual residues from either the N- or C-terminus of the protein. For *y*-type ions, which are used to identify sets of internal fragments with a common C-terminal fragmentation site, the search considers dehydrated theoretical ions. This facilitates the differentiation of gas-phase internal fragments and solution-phase protein truncations. In contrast, sets of internal fragments with a common N-terminal fragmentation site share theoretical compositions with *b*-type ions formed upon N-terminal cleavage in solution. Manual interpretation is required to distinguish between these two fragmentation events.

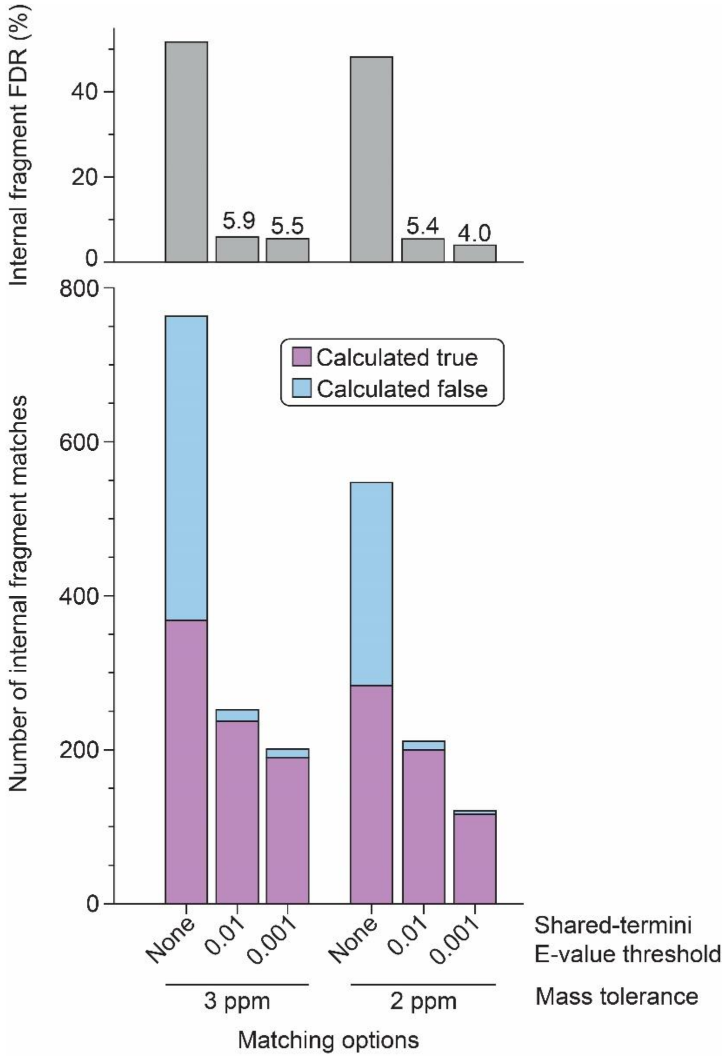

Supplementary Figure 6: precisION's multinotch enables highly effective filtering of putative internal fragments. Number of true and false internal fragments assigned with mass tolerances of either 3 or 2 ppm for the ACE2 HCD 180 V spectrum. Internal fragments were assigned using the multinotch fragment-level open search with varied significance thresholds (indicated on x axis). Ion-level discovery rates for each set of search parameters are projected above. The number of true and false fragments were determined from the calculated internal fragment ion-level FDR and the total number of assignments made. Envelope assignments were note filtered on the basis of isotopic envelope fit.

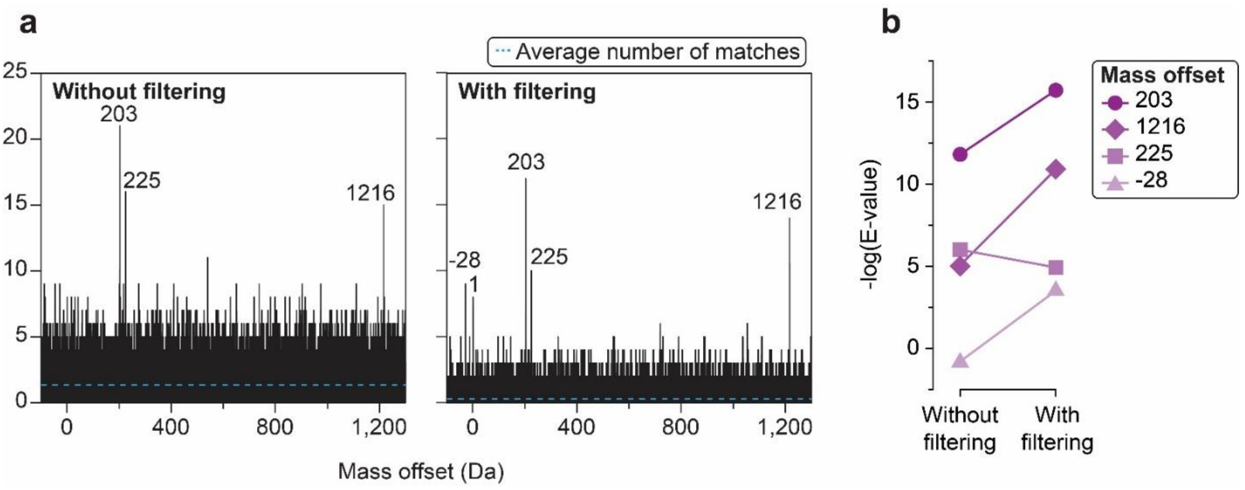

Supplementary Figure 7: Impact of the supervised voting envelope classifier on the fragment-level search. **a**, Fragment-level open search results for the ACE2 dimer (HCD 130 V) with and without filtering using precisION's envelope classifier. The average number of matches across all offsets is indicated with a dashed blue line. **b**, Statistical significance of four true mass offsets corresponding with glycosylation and CO loss, comparing results with and without envelope filtering.

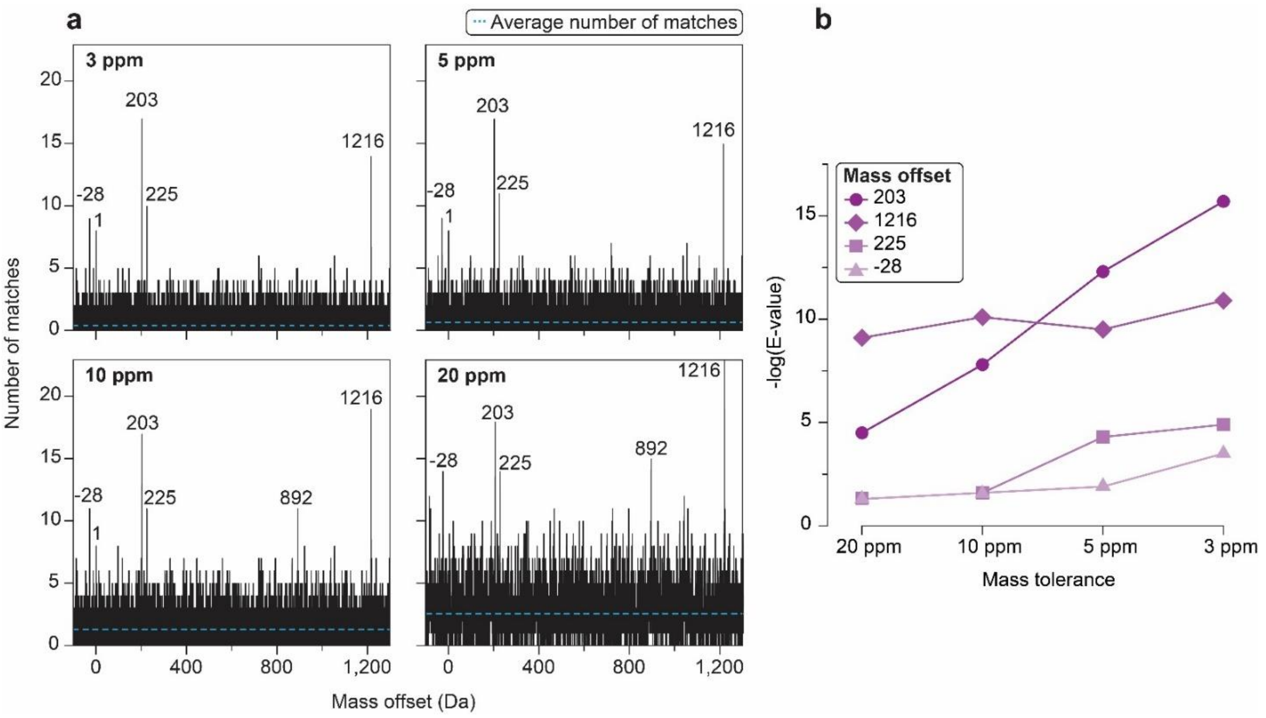

Supplementary Figure 8: Influence of the mass accuracy threshold on the fragment-level open search. a, Fragment-level open search results for the ACE2 dimer (HCD 130 V) at mass accuracy thresholds of 3, 5, 10, and 20 ppm. The average number of matches across all offsets is indicated with a dashed blue line. **b**, Statistical significance of four true mass offsets corresponding with glycosylation and CO loss. The significance of each offset is found to increase as mass tolerances are reduced.

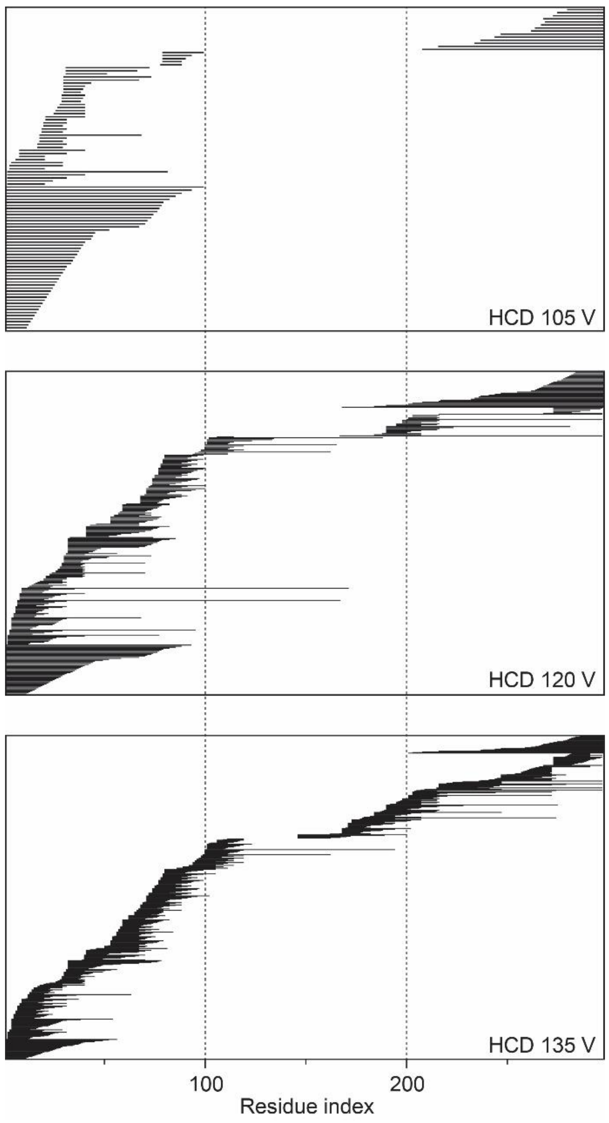

Supplementary Figure 9: Higher-energy collisional dissociation results in extensive fragmentation across the length of SPP1. Fragment maps showing the position of detected sequence ions along the sequence of SPP1 at three different HCD acceleration voltages. Individual horizontal lines correspond to assigned sequence ions. The displayed fragments are formed from the SPP1[17-314] truncation variant.

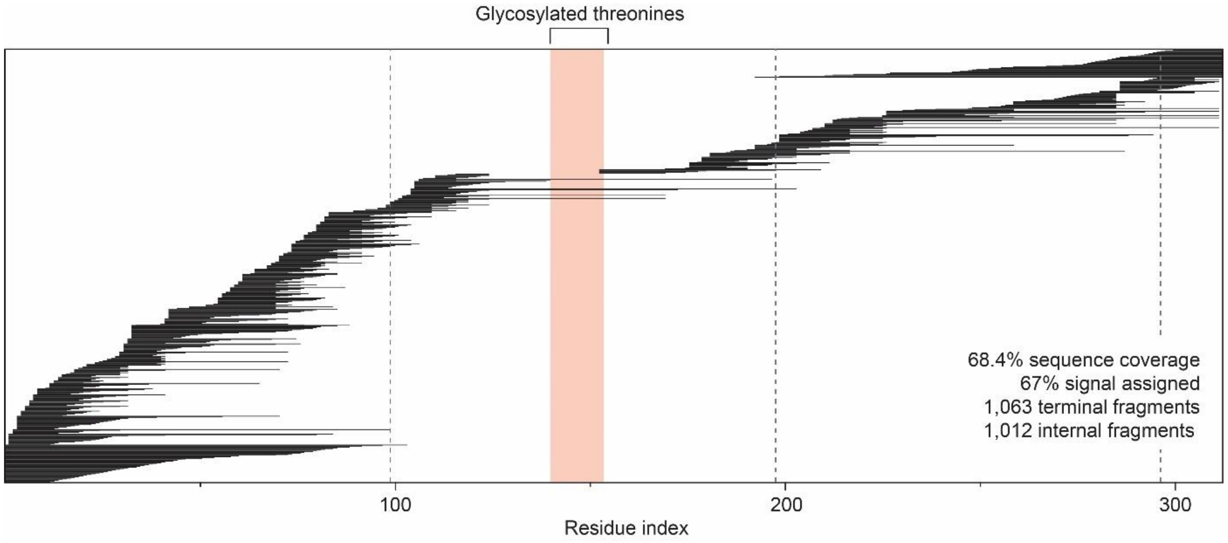

Supplementary Figure 10: Sequence ions generated by higher-energy collisional dissociation map the entire length of human SPP1. Fragment map displaying the position of the annotated fragments generated by sceHCD (105–135 V) along the sequence of SPP1. Individual horizontal lines correspond to assigned sequence ions. A region known to be extensively glycosylated is highlighted in orange—the relative lack of coverage at this site may arise due to the heterogeneity of the corresponding fragments.

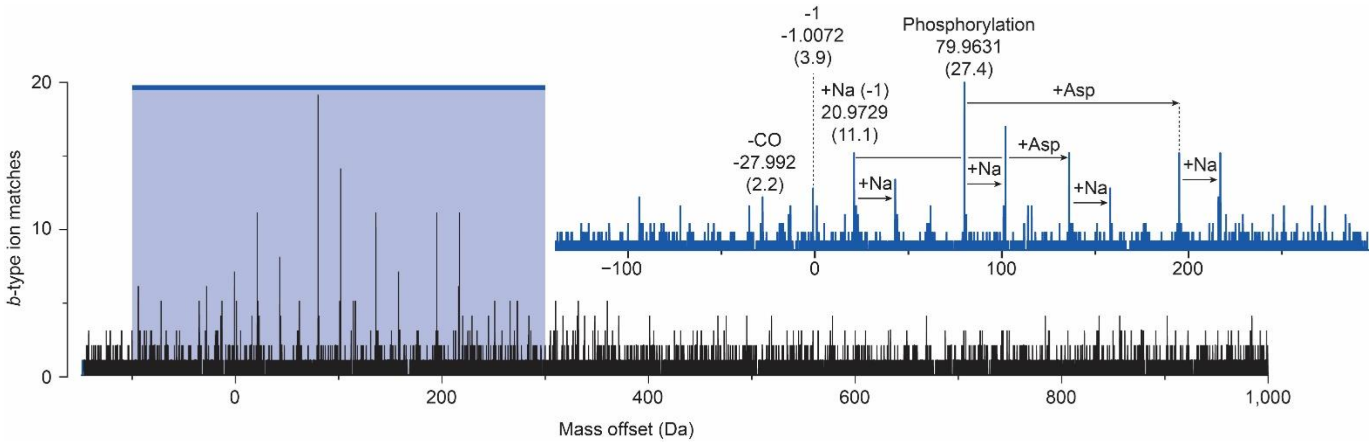

Supplementary Figure 11: A fragment-level open search reveals diverse N-terminal modification of SPP1. Full N-terminal fragment-level open search results for the SPP1 HCD 120 V spectrum. The number of *b*-type ions matched to the observed data is counted as a function of the mass offset applied to the set of theoretical ions. Measured offset masses are indicated as well as the corresponding -$\log_{10}$(E-value) in brackets.

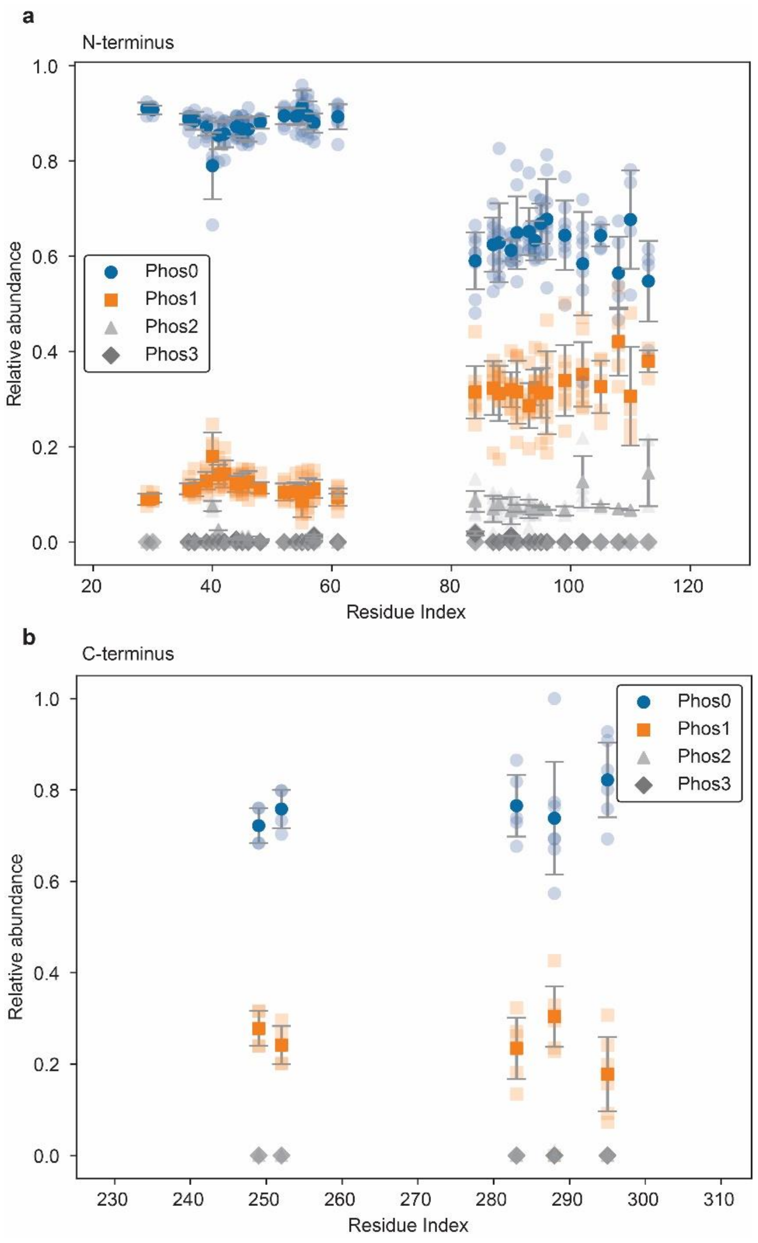

Supplementary Figure 12: Relative occupancy of different phosphate stoichiometries along SPP1. Relative abundance of sequence ions with 0 (blue circle), 1 (orange square), 2 (light grey triangles), or 3 (dark grey diamonds) phosphate groups for the **a,** N-terminus and **b,** C-terminus of SPP1[17-314]. *b*-type ions were examined for the N-terminus while *y*-type ions were examined for the C-terminus. Data are presented as mean ± s.d. from n=8 independent MS^2 spectra (acquired with different isolation windows and HCD acceleration voltages) of the same purified protein preparation. Individual data points are displayed using semi-transparent markers.

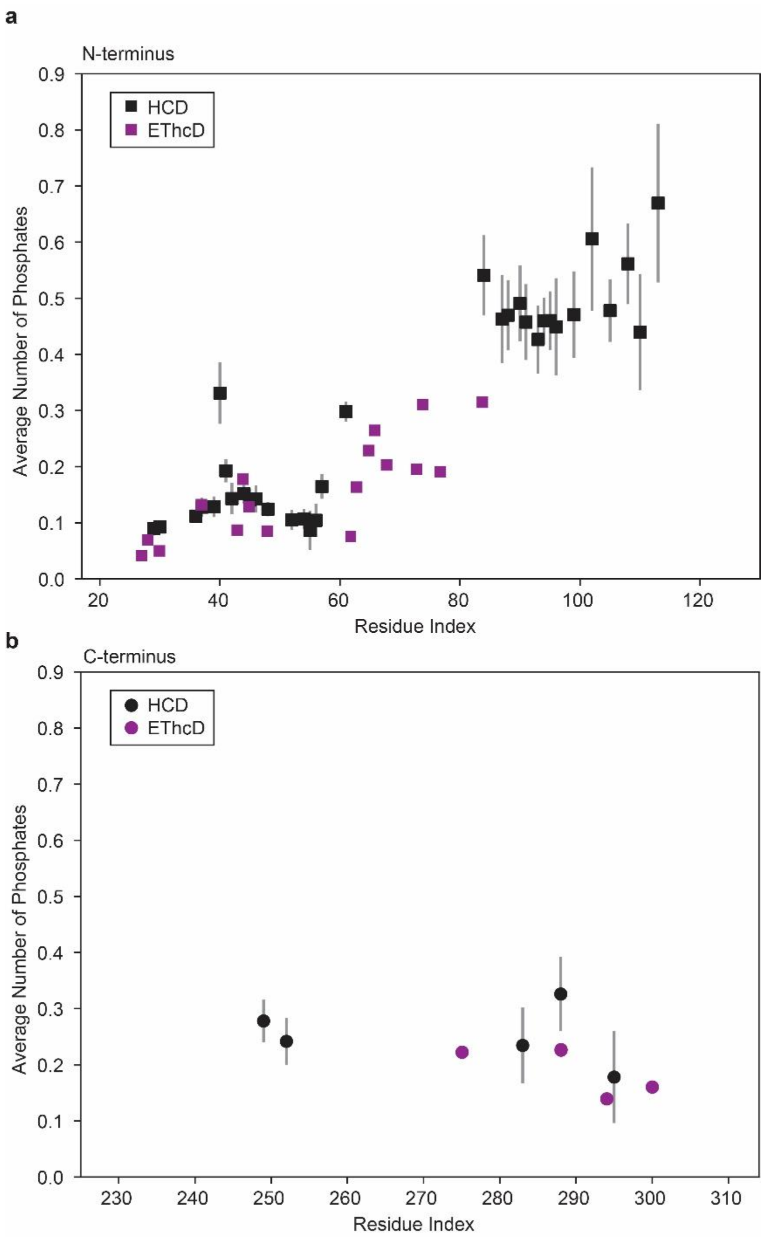

Supplementary Figure 13: Electron transfer dissociation confirms low phospho-occupancy of SPP1 termini. Comparison of MS^2-based phosphate quantification with HCD (black) and EThcD (purple) for the **a,** N-terminus and **b,** C-terminus of SPP1. For the HCD dataset, data are presented as mean ± s.d. from n=8 independent MS^2 spectra (acquired with different isolation windows and HCD acceleration voltages) of the same purified protein preparation. A single spectrum was used for the EThcD dataset. The HCD data are a direct duplicate of **Fig. 3e**.

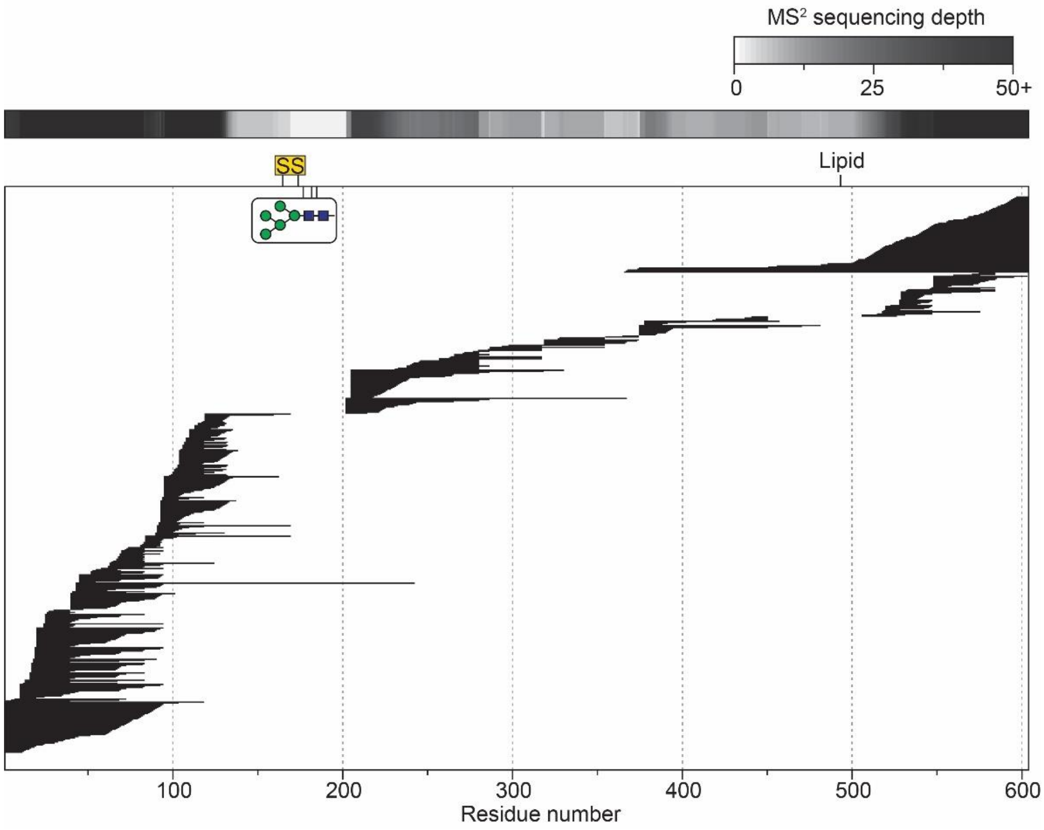

Supplementary Figure 14: Infrared multiphoton dissociation generates fragments along the length of GAT1. Fragment map displaying the position of the annotated fragments generated using slpIRMPD (6.0–8.4 W, 10 ms) along the sequence of GAT1. Individual horizontal lines correspond to sequence ions assigned from the combined slpIRMPD dataset. Known and discovered modification sites are displayed above the plot. The lack of coverage between residues 150–200 likely arises from the complex ensemble of glycans and disulfide bond at this region. An MS^2 sequencing depth plot is displayed above the fragment map. This chart illustrates the number of times each residue was observed within the combined set of fragment ions.

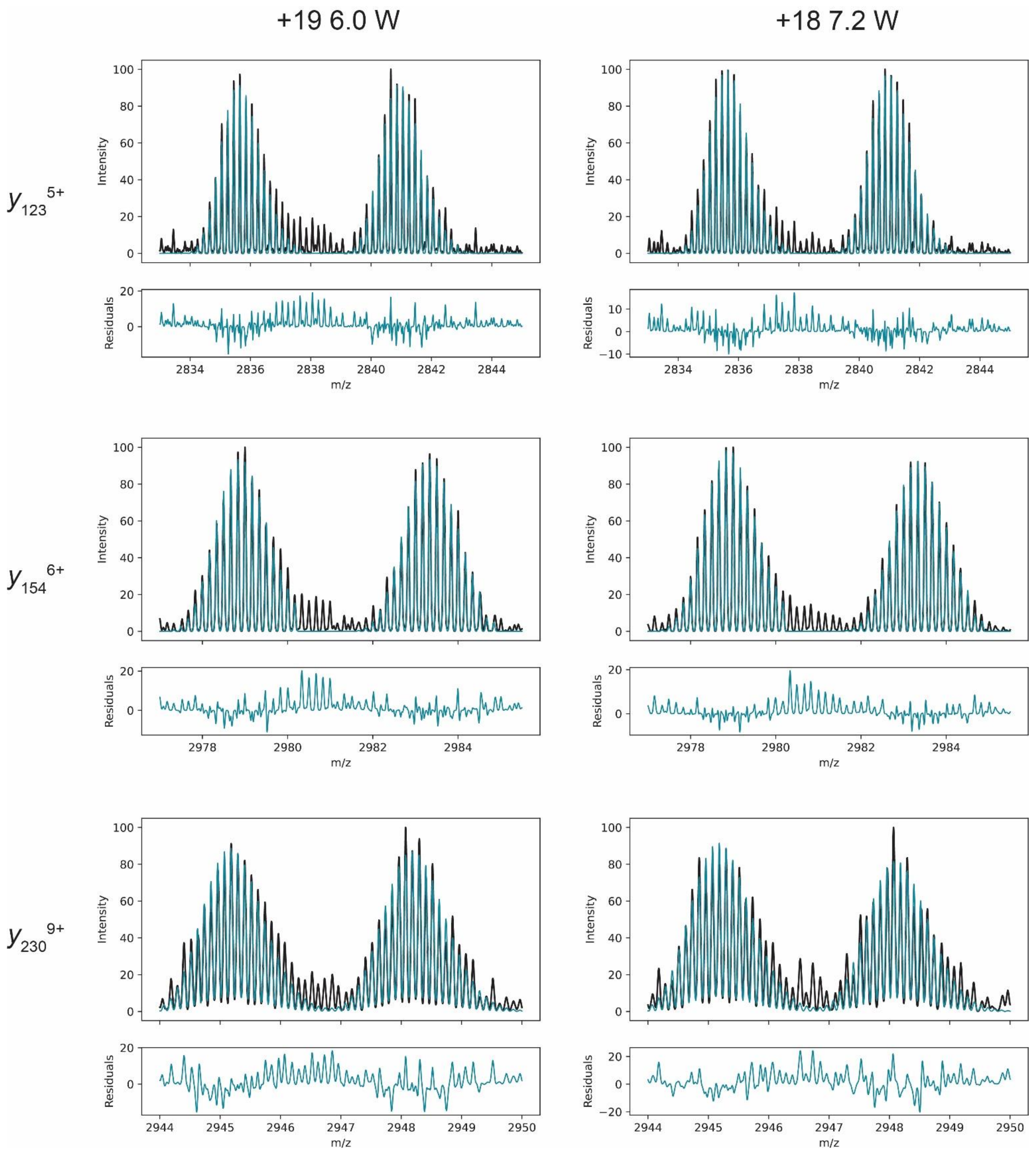

Supplementary Figure 15: Human GAT1 lipidation can be observed and reproducibly modelled across sequence ions and spectra. The observed isotopic envelopes (black) and corresponding fits of theoretical envelopes (blue) demonstrate reproducible modeling of lipid modifications (16:0, 18:0, and 18:1) on three y-type ions: Data were acquired at two laser power settings, 6.0 W and 7.2 W, each with different precursor charge states. Residual plots below each spectrum illustrate the fit quality.

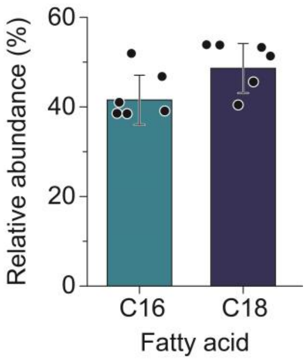

Supplementary Figure 16: Human GAT1 lipidation is consistent across replicate expressions. Bar chart illustrating the relative abundance of different fatty acids conjugated to Cys493 derived from MS^1 spectra. Data are presented as the mean ± s.d. of six independent GAT1 samples isolated from HEK293 GNTI$^{-/-}$ cells with differing passage numbers.

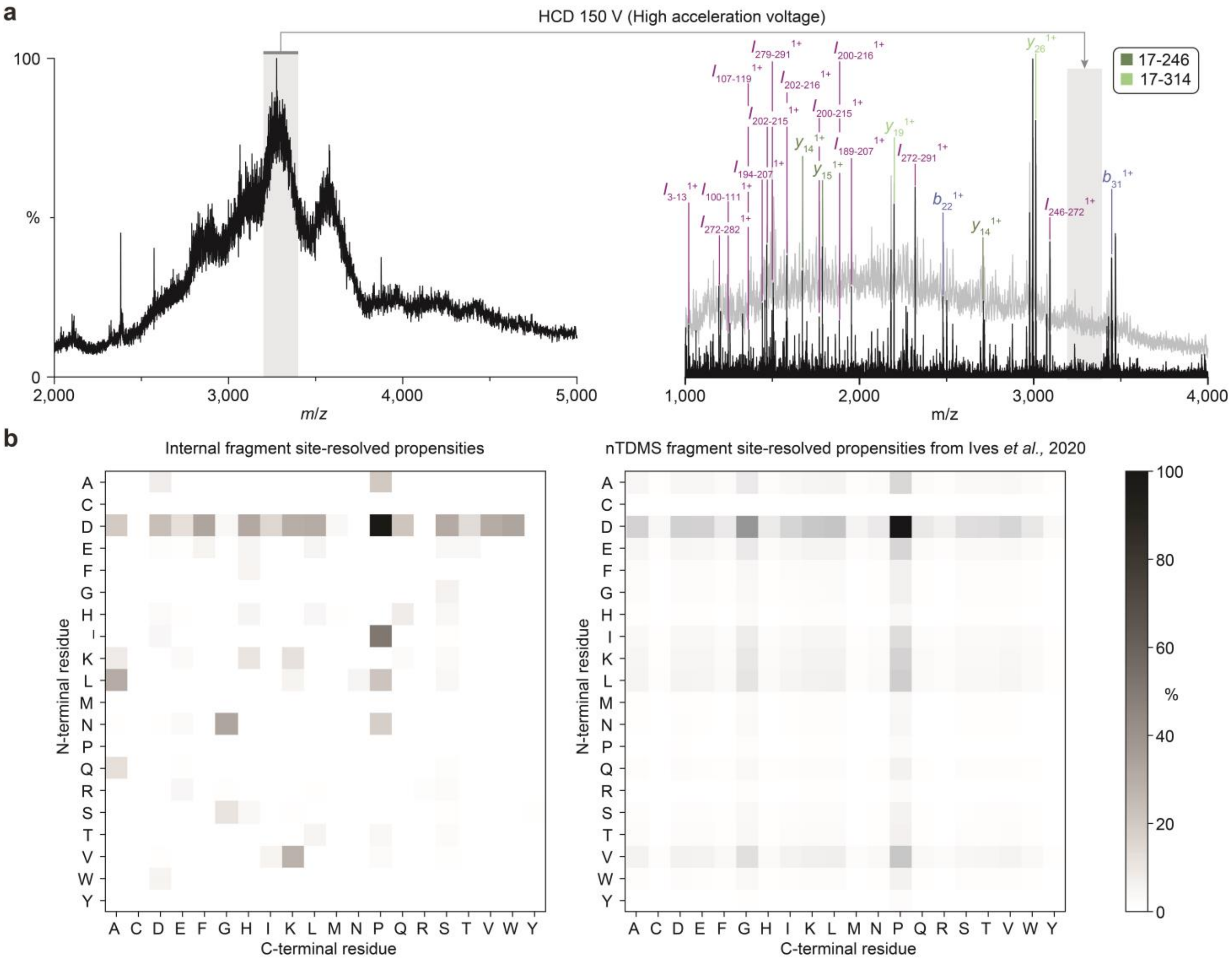

Supplementary Figure 17: Internal fragments in nTDMS primarily form *via* bond cleavage at high-propensity fragmentation sites. a, Mass spectrum of SPP1 (1 M ammonium acetate, pH 7.0). A range of ions (m/z 3,300 ± 100) were selected using the quadrupole and activated using ion–neutral collisions (HCD 150 V). The annotated MS^2 spectrum is displayed to the right. Two truncated forms of the protein are annotated in different shades of green. Internal fragments are annotated in purple. The set of fragment ions form a dense array across the *m*/*z* domain, as highlighted by the ion trap spectrum (grey trace) which displays an elevated baseline. **b,** Comparison of the site-resolved fragmentation propensities derived from the SPP1 HCD 150 V dataset (internal fragments only), and a large-scale study of native protein fragmentation that examined terminal fragments for a range of protein complexes (Ives *et al.*, 2020).

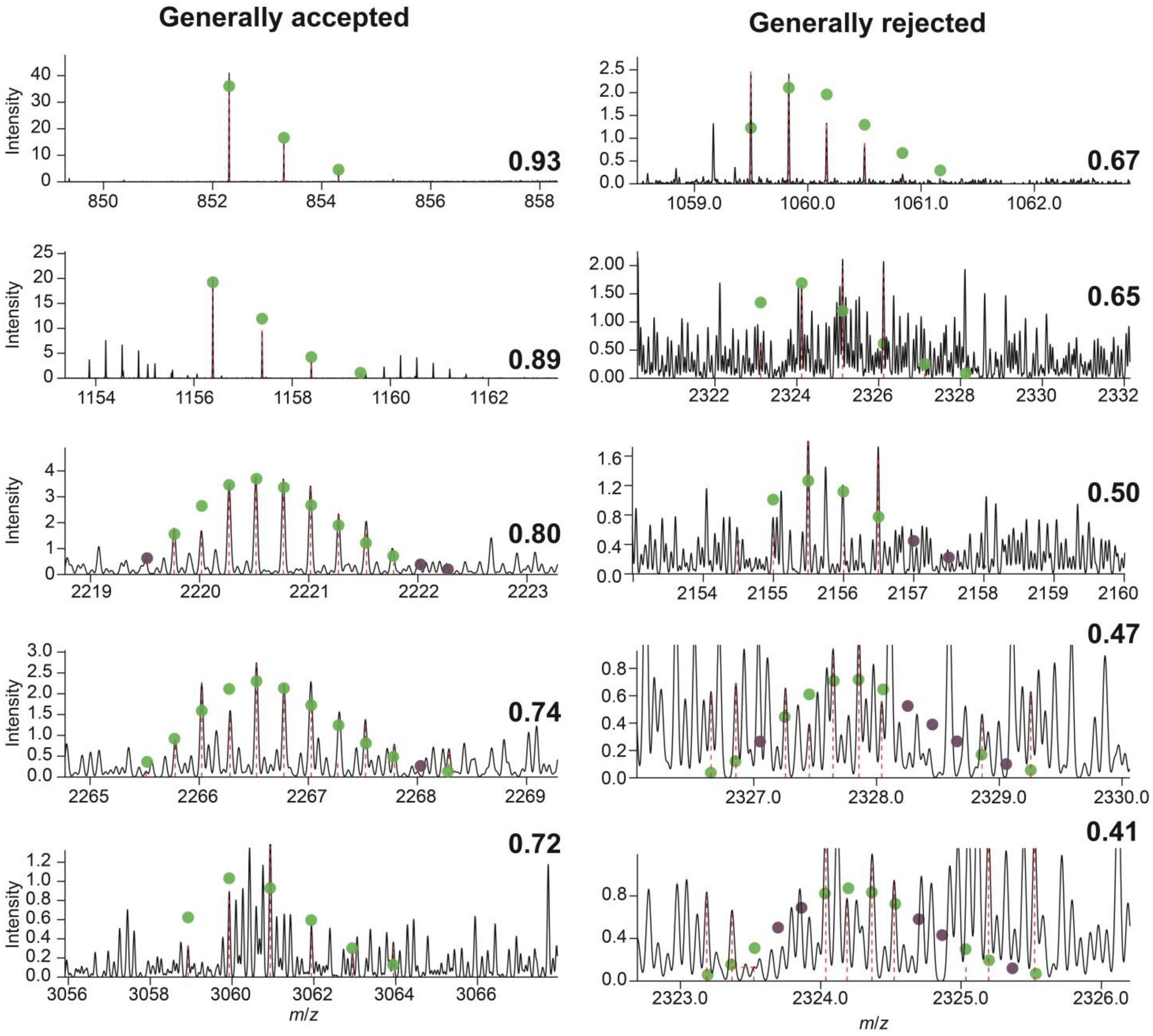

Supplementary Figure 18: True and false fragment ions can be partially distinguished on the basis of their isotopic envelope fit. Example isotopic envelopes with fit scores ranging from 0.93 to 0.41. Theoretical envelopes are displayed with green (peak detected) or purple (peak not detected) dots. Typically, ~90% of assigned envelopes exhibit fit scores greater than 0.70.

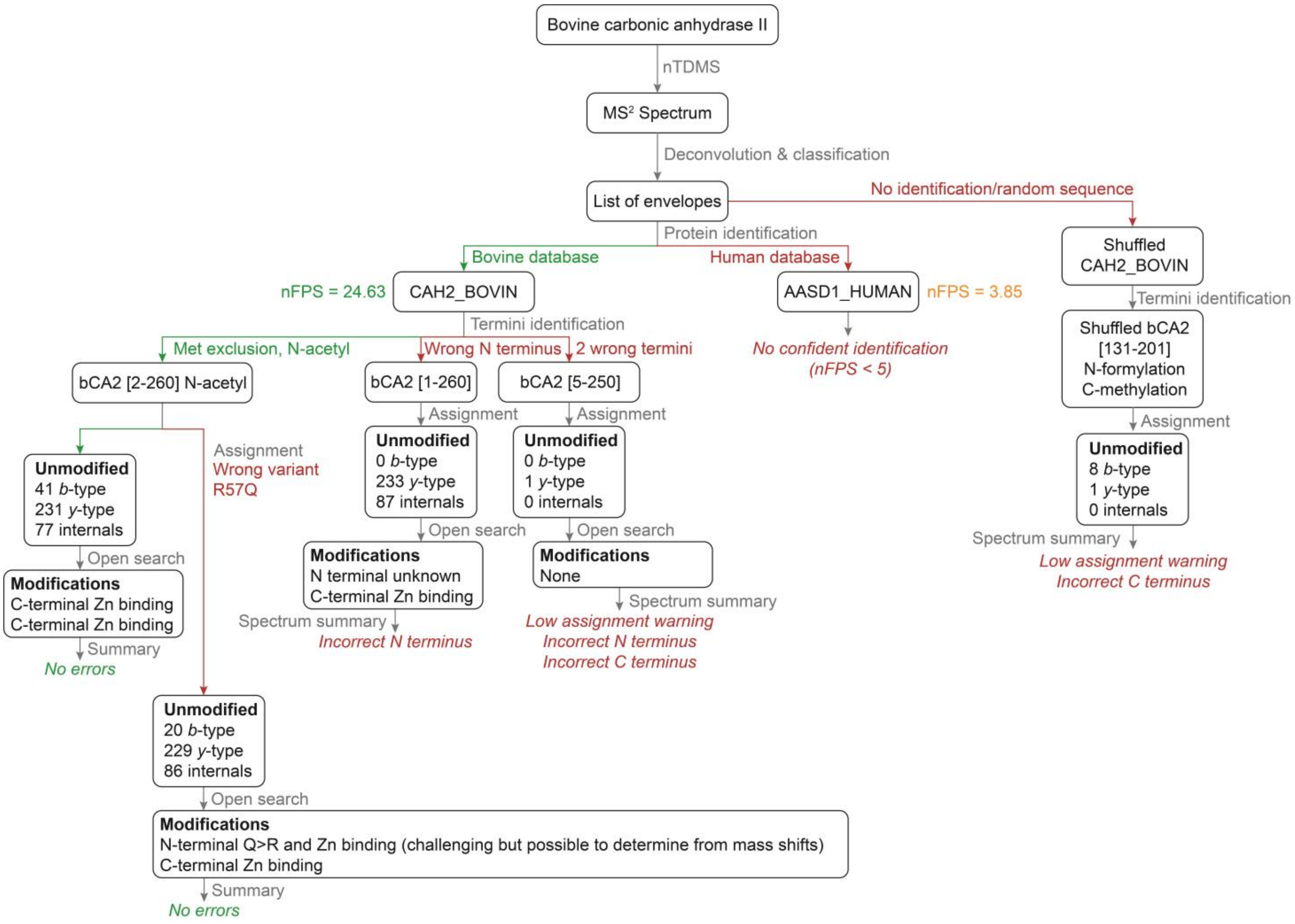

Supplementary Figure 19: precisION correctly identifies misassignments in entrapment-style evaluations. Flow chart representing the findings from an entrapment-style evaluation of precisION. Random sequences, incorrect source organisms, and incorrect termini were correctly identified by the software package. When an incorrect variant was considered, the fragment-level open search was used to identify and correct the mutation. Full details of the entrapment-style analyses are provided in **Supplementary Note 2.** nFPS, native fragmentation propensity score.

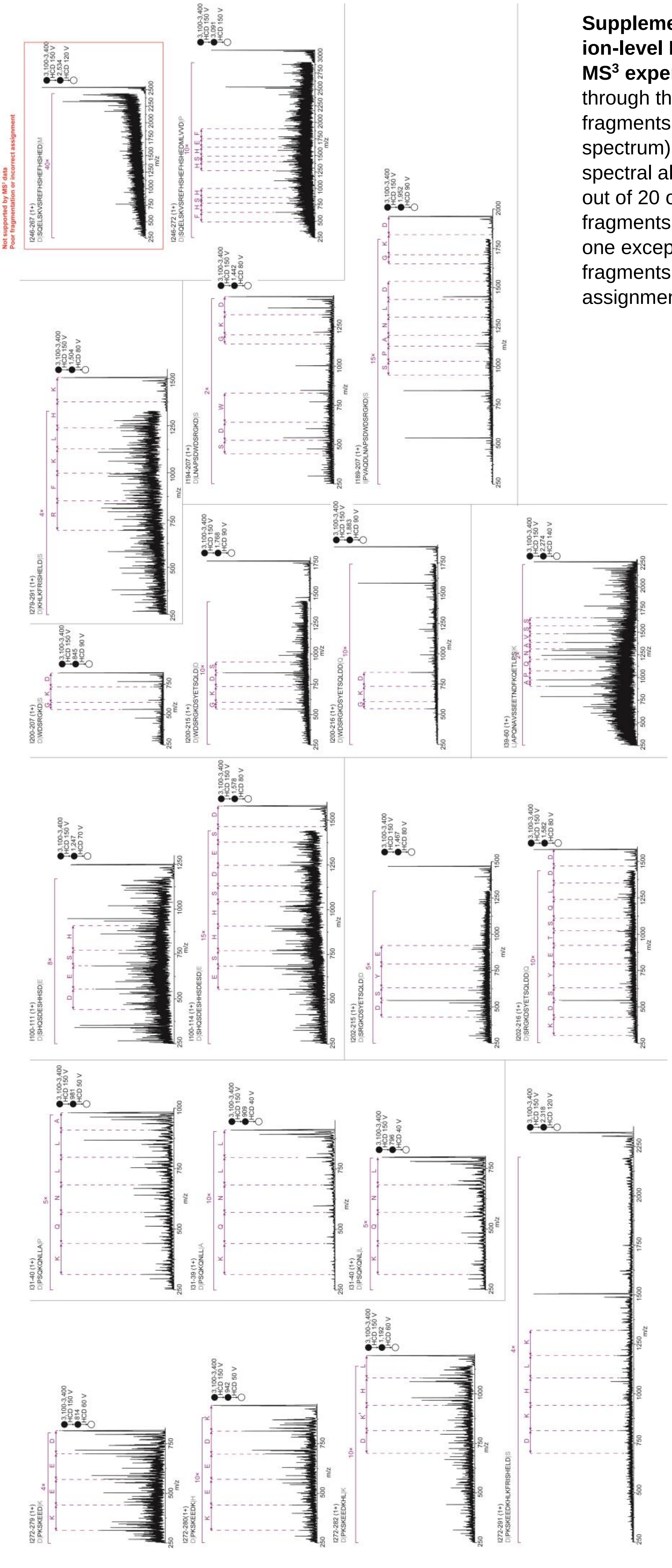

Supplementary Figure 20: Internal fragment ion-level FDR calculations are supported by MS^3 experiments. Twenty MS^3 spectra acquired through the activation of putative internal fragments (see **Supplementary Fig. 16** for MS^2 spectrum). Ion identities were confirmed using spectral alignment and *de novo* sequencing. 19 out of 20 of the evaluated putative internal fragments were supported by the MS^3 data. The one exception did not produce any informative fragments for *de novo* sequencing, so no alternate assignment could be proposed.

Supplementary Table 1: List of features used to represent putative fragment ion envelopes

	Feature	Description
1	Charge	Charge of the fragment ion
2	Fit score	Custom goodness-of-fit score described in **Supplementary Note 3**
3	Interference score	Proportion of signal within the span of the fragment ion's isotopic envelope that is not assigned to the fragment ion
4	log(S/N)	Common logarithm of the signal-to-noise ratio where the noise level is defined as the modal peak intensity
5	% Missing peaks	Percentage of the theoretical isotopologue peaks that could not be detected.
6	stddev(Mass errors)	Standard deviation of the mass errors for each detected isotopologue
7	χ^2	Test statistic from a chi-square goodness-of-fit test comparing the theoretical and observed isotopologue intensities
8	χ^2 *p*-value	*p*-value from a chi-square goodness-of-fit test comparing the theoretical and observed isotopologue intensities. H_0: The observed ions come from the theoretical distribution of isotopologues
9	PCC	Pearson correlation coefficient measuring the linear correlation between the theoretical and observed isotopologue intensities
10	Pearson correlation *p*-value	*p*-value from Pearson correlation analysis. H_0: The observed relationship between the theoretical and observed isotoplogue intensities is due to chance.

Supplementary Table 2: List of SPP1 MS/MS spectra used for the quantification of protein phosphorylation

Isolation window	HCD acceleration voltage (v)
2,900–3,100	70
	80
3,100–3,300	70
	80
3,300–3,500	80
	95
3,500–3,700	90
3,700–3,800	95

Supplementary Note 1

To demonstrate the applicability of precisION to the analysis of endogenous multiprotein complexes, we investigated the composition of the heteromeric PDE6 complex isolated from bovine retina. Native MS alone revealed an intact complex with a molecular weight of 217,750 Da, in approximate agreement with the expected mass of 217,370 Da. However, extensive adduction resulted in broad peaks in the deconvolved spectrum (FWHM = 940 Da), obfuscating potential post-translational modifications (**Fig. 1a**). To overcome this limitation and uncover hidden modifications, we selected the 30+ charge state (*m*/*z* 7250 ± 5) for activation with IRMPD (**Extended Data Fig. 1**). We subsequently analyzed the resulting sequence ions using precisION, confirming the identity of the heteromeric complex based on the strong matches with all three mature subunits of the complex (**Extended Data Fig. 1a**). Each subunit was found to be N-terminally acetylated (**Extended Data Fig. 1b**), with a fragment-level open search confirming C-terminal geranylgeranylation of PDE6β (**Extended Data Fig. 1c**). Intriguingly, we could not detect *S*-farnesylation of PDE6α's C-terminal cysteine residue. To deduce why, we used precisION's inbuilt visualization module to map fragment ion intensities across the protein sequence. Such analysis revealed preferential fragmentation from the N terminal regions of the α and β subunits (**Extended Data Fig. 1d,e**), likely due to localized charge enrichment. The relative scarcity of C-terminal fragmentation likely accounts for the absence of detectable *S*-farnesylation. Together, these results demonstrate that the endogenous PDE6 complex undergoes extensive and complete post-translational modification. The observed modifications are consistent with a role in complex assembly and functional maturation *in vivo*.

Supplementary Note 2

precisION is designed to attempt identifications from MS^2 spectra under a wide range of conditions, including cases where the available evidence may be limited or ambiguous. While this flexibility allows users to recover identifications from challenging datasets, it also raises the possibility of unsupported or erroneous assignments, especially if incorrect protein sequences are used early in the workflow. Such early errors may propagate through downstream steps, for example during recalibration. To address this challenge, we have implemented a set of quantitative metrics, qualitative diagnostics, and automated warning flags in precisION to help users detect and interpret uncertain results. These measures are intended to complement clear visualizations of the data, such as annotated spectra, fragment maps, calibration curves, fragmentation propensity heatmaps, and 3D structure maps.

To assess the robustness of these safeguards, we conducted an entrapment-style evaluation of the software, using a MS/MS spectrum acquired through HCD activation of native bovine carbonic anhydrase II (**Supplementary Fig. 19**). The aim of this evaluation was to explore how precisION behaves when faced with varying levels of incorrect information and to determine how effectively the software flags and reports these errors.

1. **Shuffled wild-type sequence:** A shuffled version of the wild-type protein sequence was considered, and the termini that yielded the highest number of fragment matches were selected, despite their low biological plausibility. Unmodified sequence ions were then assigned, and the full workflow (including recalibration and open search) was executed without manual intervention. No significant mass offsets were detected during the fragment-level open search, indicating no support for modifications or mutations. Upon evaluation of the results, two automated warning flags were raised indicating that the analyzed data should be interpreted with caution.
2. **Incorrect database:** We looked for matches to the spectrum in the human proteome. However, no protein afforded a native fragmentation propensity score (nFPS) greater than the “green” threshold of 5, demonstrating that no confident assignment could be made.
3. **Incorrect termini:** We manually selected the incorrect termini, despite clear results from the multinotch fragment-level open search. In such cases, precisION was able to identify which termini were incorrect.
4. **Incorrect variant:** When a known variant of carbonic anhydrase II was considered (R57Q), the fragment-level open search returned a significant delta mass (+90.9566 Da) on *b*-type ions. This mass shift was attributable to the Q>R mutation and Zn binding, demonstrating that precisION was able to guide the user toward the correct variant, despite the presence of a combined, complex modification. While such a case may be difficult to resolve blindly, the open search made it evident that additional modifications or mutations needed to be considered.

In all cases, precisION was able to flag incorrect assignments, demonstrating the resistance of the analysis pipeline to incorrect assignments, even in the case of early-stage errors.

Supplementary Note 3

precisION uses a custom goodness-of-fit score that prioritizes the fit of high-abundance isotopologues.

Theoretical envelopes are fit to the data by varying *k* to minimize the sum S,

$$S = \sum_{i=0}^{n} \left(\left(k \cdot y_{theo_i} - y_{exp_i} \right) \times y_{theo_i} \right)^2$$

where y_{theo} and y_{exp} are the vectors of theoretical and observed isotopologue intensities, respectively.

The goodness-of-fit score is then calculated as follows,

$$\frac{\sum_{i=0}^{n} k \cdot y_{theo_i} \times min\left\{ \frac{y_{exp_i}}{k \cdot y_{theo_i}}, \frac{k \cdot y_{theo_i}}{y_{exp_i}} \right\}}{\sum_{i=0}^{n} k \cdot y_{theo_i}}$$

Pearson correlation analysis and chi-square goodness-of-fit tests are conducted after fitting.
